# Prognostic significance of huntingtin interacting protein 1 expression on patients with acute myeloid leukemia

**DOI:** 10.1038/srep45960

**Published:** 2017-04-28

**Authors:** Jinghan Wang, Mengxia Yu, Qi Guo, Qiuling Ma, Chao Hu, Zhixin Ma, Xiufeng Yin, Xia Li, Yungui Wang, Hanzhang Pan, Dongmei Wang, Jiansong Huang, Haitao Meng, Hongyan Tong, Wenbin Qian, Jie Jin

**Affiliations:** 1 Department of Hematology, The First Affiliated Hospital, Zhejiang University College of Medicine, Hangzhou, China; 2 Institute of Hematology, Zhejiang University, Hangzhou, China; 3 Key Laboratory of Hematologic Malignancies, Diagnosis and Treatment, Zhejiang, Hangzhou, China; 4 Department of Nephrology, The First Affiliated Hospital, Zhejiang University, Hangzhou, China; 5 Department of Hematology, The Second Affiliated Hospital of Henan University of Traditional Chinese Medicine, Zhengzhou, China

## Abstract

Huntingtin interacting protein 1 (HIP1) is an endocytic protein which is overexpressed in a variety of human cancers and involved in cancer-causing translocation in leukemia. However, the prognostic impact of *HIP1* expression on AML remains unclear. In this study, quantification of *HIP1* transcript by real-time quantitative PCR in bone marrow blasts was performed in 270 AML patients. As a result, high *HIP1* expression was seen more frequently in older patients, M4/M5 morphology and genes of *NPM1* and *DNMT3A* mutations, and underrepresented in favorable karyotype subgroups and *CEBPA* double allele mutations in our AML patients. We also found high *HIP1* expressers showed lower levels of hemoglobin. In addition, overexpression of *HIP1* was associated with an inferior overall survival. The prognostic value of *HIP1* expression was validated in patients from an independent TCGA cohort. Notably, up-regulation of miR-16, miR-15a, miR-28 and miR-660 were seen in high *HIP1* expressers from the two independent cohorts. *In vitro*, interfereing of *HIP1* expression by siRNA suppressed the proliferation of leukemic cells, and downregulation of these miRNAs were seen in THP-1 and Kasumi cell lines after silencing *HIP1* expression. In conclusion, the *HIP1* gene expression might serve as a reliable predictor for overall survival in AML patients.

Acute myeloid leukemia (AML) is a heterogeneous group of hematologic malignancies with various genetic abnormalities and variable responses to treatment. To date, AML patients can be classified into three risk subgroups according to karyotype abnormalities: favorable, intermediate and adverse. In addition to chromosome lesions, several genes such as *NPM1, FLT3*-ITD and *CEBPA* mutations have been recommended as reliable prognostic factors^1^. However, only half of AML patients obtained cytogenetic abnormalities. Thus, reliable biomarkers are still required in clinical practice^2^.

With the advent of the high throughput transcriptomic profiling, biomarker identification has been taken to the genomic level^3^. Although multiple genes, particularly signaling pathways, provide a stronger and more reliable prognostic assessment, prognostic effects must first be studied at the individual gene level. This is because such an analysis will provide a rationale for mechanistic studies followed by therapeutic targeting. The hypothesis is that one disrupted gene was enough to regulate the relevant signaling pathway, leading to leukemia cell proliferation or metastasis. Thus, in order to identify such a driver gene, we searched for public articles about pathway analysis using gene microarray for AML patients. Fortunately, we found that huntington’s disease (HD) signaling pathway is one of the most significant pathways changed in AML blasts compared with normal CD34 bone marrow samples as previously reported^4^. We therefore focused on the HD signaling pathway to uncover the underlying oncogene. It is believed that mutant huntingtin (HTT) protein causes HD. One of the mechanisms is mutant HTT protein leads to decreased binding affinity for HTT interacting protein 1 (HIP1), thus causing disruption of HIP1’s normal function, and also accumulation of high levels of the free form of HIP1^5^. HIP1 contains evolutionarily conserved sequences, including a leucine zipper motif and a carboxyl terminus with homology to TALIN, a cytoskeletal actin binding protein^6^. Although the true function of HIP1 remains unknown, it has been shown HIP1 protein has a role in the clathrin-mediated endocytosis which regulates several different signaling pathways, receptor trafficking and cytoskeleton dynamics^7^. Notably, it has previously reported alterations in HIP1 protein have been associated with tumors. Analysis by western blot showed more than 50 cancer cell lines had high levels of HIP1 protein^7^. Similarly, overexpression of *HIP1* gene was also observed in multiple human cancers including prostate cancer^8^, breast cancer^9^, brain tumor^10^, Merkel cell carcinomas^11^ and lymphoma^12^. Furthermore, *in vitro* analysis of the effects of *HIP1* overexpression on cells indicated that it can transform fibroblasts^9^. These results suggest *HIP1* acts as an oncogene in solid tumors. With respect to hematopoietic malignancies, the first clue that *HIP1* might have a role in tumorigenesis came in 1988, when the fusion of HIP1 and platelet-derived growth factor receptor was discovered as the cause of a chronic myelomonocytic leukemia^13^. Taken together, *HIP1* expression might serve as a useful biomarker in AML owing to the oncogenic propensity. However, the biological feature and prognostic value of *HIP1* expression in AML blasts remains unclear.

Here, we found AML patients with high *HIP1* expression had a distinct microRNA signature and poor survival in our large cohort of patients. The prognostic value of *HIP1* expression was also validated in an independent cohort of AML patients. This study provides a reliable prognostic biomarker and critical drug target for AML patients.

## Results

### Characteristics of patients with high *HIP1* expression

The distribution of *HIP1* expression was binormal and exhibited two clusters low and high expressers (Figure S1). The cutoff value was estimated using Cutoff Finder software analysis. Thus, 90 (33%) were classified as low and 180 (67%) high *HIP1* expression. Clinical characteristics of patients with high *HIP1* expression are summarized in Table 1. High expressers were older (P = 0.013), had lower hemoglobin levels (P = 0.026), and were more frequently in AML FAB subtype M4 (12% vs. 3%) and M5 (28% vs. 18%) morphology (P = 0.025) than low expressers. Patients with high *HIP1* expression were associated with a significantly higher frequency of *NPM1* mutations (30% vs. 17%, P = 0.036) and *DNMT3A* mutations (13% vs. 5%, P = 0.031), a significantly lower frequency of favorable karyotype risk subgroup (2% vs. 9%, P = 0.037), *CEBPA* double allele mutations (7% vs. 24%, P < 0.001), compared with patients with low expression. There was no statistically significant correlation between *HIP1* expression and other variables including sex, white blood cell counts (WBC), platelet counts, percentage of bone marrow blasts and *FLT3-*ITD positive and treatment protocols (Table 1).

### Association of *HIP1* expression with clinical outcome from the ZIH cohort

**Figure 1:**
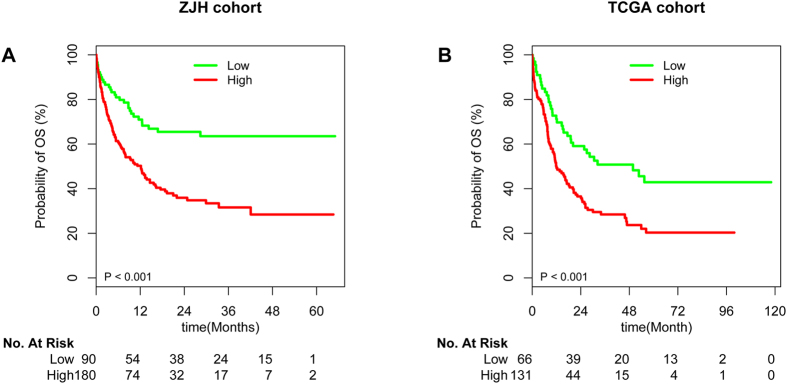
Survival curves of AML patients. Kaplan-Meier estimates of OS by high and low *HIP1* expression for our patients (**A**) and patients from the TCGA cohort (**B**), respectively.

With a median follow-up for living patients of 484 days with 95% confidence interval 374–1262 days, high *HIP1* expressers (n = 180) had more adverse OS compared to low expressers (n = 90) (Fig. 1A). Importantly, in the subgroup analyses we found high *HIP1* expressions were associated with poor OS in patients with both cytogenetic intermediate risk group and cytogenetically normal AML (Figure S2A,B). In order to identify the potential confounders or interactive factors, we conducted stratified analyses and interactive analyses. As shown in Supplementary Figure S3, there were no significant interactions among these factors. Even if we taken these factors as confounders, *HIP1* expression was still as an independent prognostic factor in multivariate analysis after adjusting for age, WBC, karyotype risk groups, and genes of *FLT3-*ITD, *NPM1, CEBPA* and *DNMT3A* mutations [for OS HR (95%CI), 1.658 (1.068, 2.576); P = 0.024; Table 2]. Moreover, we also conducted landmark analysis by including patients whose survival was more than 30 days in order to ignore the cause of induction death by intense chemotherapy. As a result, high expression of *HIP1* was still independently associated with poor OS [HR (95%CI), 1.766(1.074, 2.905), P = 0.025] in the multivariate survival analyses (Table S1).

With respect to the induction remission rate, high expressers had lower complete remission rate compared with low expressers in univariate analysis [OR (95%CI), 0.491(0.256,0.903), P = 0.026, Table S2]. However, the significance did not stand after adjustment with other factors like age, WBC, cytogenetic risk groups, genes of *FLT3-*ITD, *NPM1, CEBPA* and *DNMT3A* mutations and treatment protocols in the multivariate analysis (Table S2).

### Validation of the impact of *HIP1* expression on survival from the TCGA cohort

In order to validate the prognostic values of *HIP1* expression in our cohort, we defined low *HIP1* expressers from TCGA cohort using the same percentage (33%) of low expressers based on the same estimated method as mentioned in our cohort (Figure S1B). Correspondingly, of 197 patients from the TCGA cohort, 66 (33%) were defined as low *HIP1* expressers and 131 (67%) as high expressers. Characteristics of patients with high *HIP1* expression from the TCGA cohort were described in Supplementary Table S3. Similarly, high *HIP1* expressers were predominant in older patients (P = 0.04), more frequently in M4 (29% vs. 5%) and M5 (15% vs. 5%) morphology (P < 0.001), had a significantly higher frequency of *NPM1* mutations (36% vs.11%, P < 0.001) and *DNMT3A* mutations (32% vs. 12%, P = 0.003), and had a significantly lower frequency of favorable karyotype risk subgroup (11% vs. 33%, P < 0.001), compared with low expressers (Table S3). In contrast with our cohort, we also found high expressers had higher levels of WBC and a higher frequency of *FLT3*-ITD positive. There are no differences between high and low expressers with respect to sex, percent BM blasts, genes of *CEBPA, IDH1, IDH2* mutations and consolidation therapy such as bone marrow transplantations.

To test whether HIP1 was a robust biomarker among gene expression profiles of AML in the TCGA cohort, we carried out the resampling statistics using the method of multiple survival screening (MSS)^14^. As a result, 45 genes whose q-values are less than 0.05 were identified as survival genes. As expected, HIP1 was observed as a robust biomarker in the top 30 most frequent genes among the predictive random gene sets (Table S4). In univariate analysis, patients with high *HIP1* expressers were associated with adverse OS compared with lower expressers (Fig. 1B). Moreover, in multivariate analysis, high *HIP1* expressers were significantly associated with poor OS [HR, 1.558(1.017, 2.385); P = 0.041, Table S5] in the context of age, WBC, karyotype-risk groups and genes mutations of *FLT3-*ITD, *NPM1, CEBPA, DNMT3A, IDH1, IDH2*, and bone marrow transplantation.

### MicroRNA expression profiling

**Figure 2:**
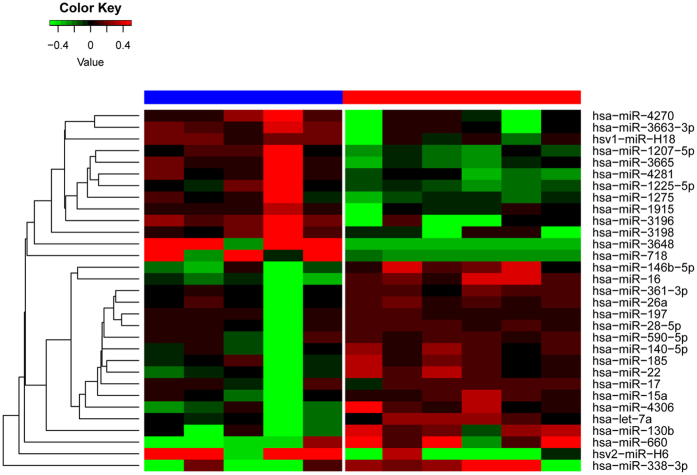
Heatmap plot illustrating the microRNAs expression between high and low *HIP1* expression.

**Figure 3:**
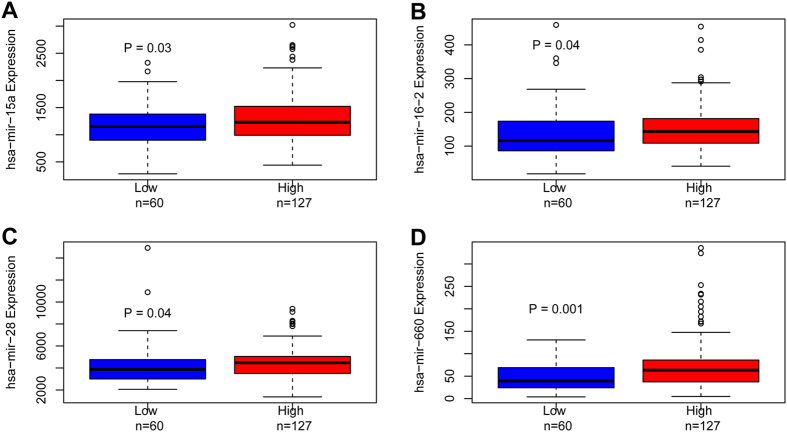
Validation of microRNAs expression in patients with low vs high *HIP1* expression in TCGA cohort.

We applied the propensity score analysis to match each CN-AML patient with high and low *HIP1* in our cohort, matching was based on age, WBC, cytogenetic group and genes of *FLT3-*ITD, *NPM1, CEBPA, DNMT3A* mutations which might affect the microRNA expression (Table S6). Thus, we selected six samples with high and five matching samples with low *HIP1* expression to assess the differences of microRNA (miR) expression. The most significant changes of miRs in high expressers included up-regulation of miR-146b-5p, miR-16, miR-361-3p, miR-26a, miR-197, miR-28-5p, miR-590-5p, miR-140-5p, miR-185, miR-22, miR-17, miR-15a, miR-4306, let-7a, miR-130b, miR-660 and miR-338-3p and down-regulation of miR-4270, miR-3663-3p, hsv1-miR-H18, hsv2-miR-H6, miR-3665, miR-1225-5p, miR-1275, miR-1915, miR-3196, miR-3198, miR-3648 and miR-718 (p-value < 0.05, Fig. 2). Among these miRs, up-regulation of miR-16, miR-15a, miR-28 and miR-660 were also seen significantly changes in high *HIP1* expressers in a large and independent cohort of TCGA patients (Fig. 3).

### siRNA interference

**Figure 4:**
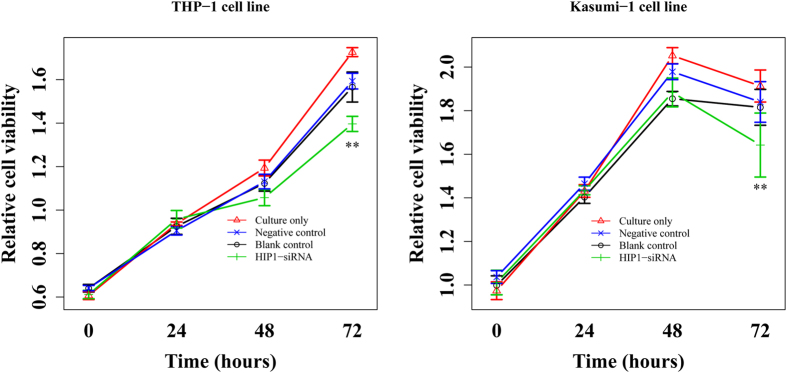
Proliferation of THP-1 and Kasumi-1 cells in the different transfected condition.

**Figure 5:**
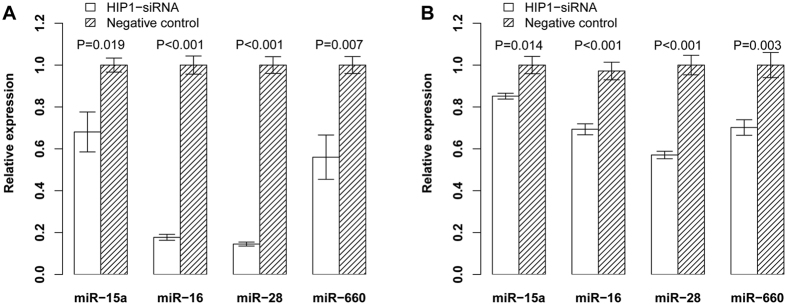
Measurement of the expression of miR-15a, miR-16, miR-28-5p and miR-660 in THP-1 (**A**) and Kasumi-1 (**B**) cell lines transfected with HIP1-siRNA and negative control siRNA.

The relevance of high levels of *HIP1* expression to the poor survival suggests that *HIP1* may be functionally important for maintaining the continuous proliferation of leukemia cells. To example this possibility, we measured the proliferation of Kasumi-1 and THP-1 cells using siRNA to silence *HIP1* expression. Quantitative RT-PCR analysis showed that siRNA treatment resulted in approximately 83% and 63% knockdown of *HIP1* mRNA expression in THP-1 and Kasumi-1 leukemia cells after 72 hours compared to negative control (Figure S4). It also led to a significant decreased growth of THP-1 and Kasumi-1 cells (Fig. 4). Importantly, *HIP1* interference in THP-1 cell line dramatically reduced the expression of miR-16, miR-15a, miR-28 and miR-660 (Fig. 5A). In parallel, silencing *HIP1* expression in Kasumi-1 cell line significantly reduced the expression of these miRNAs (Fig. 5B). These results suggested that *HIP1* might offer a valuable therapeutic target.

### Integrative analysis of mRNA and miRNA interaction between high and low *HIP1* expressers

We analyzed the gene expression patterns of leukemia blasts from 131 patients with low expression and 66 patients with high expression from the published TCGA data^15^. We found 475 genes were down-regulated and 662 genes were up-regulated in high expressers (Figure S5). By means of miRNA-mRNA integrative analysis, we found several targeted genes of miR-28-5p, miR-15a, miR-16 and miR-660. Specifically, among these 1137 aberrantly expressed genes, 84 genes were predicted to be targeted by miR-28-5p, 100 by miR-15a, 100 by miR-16 and 58 by miR-600 (Figure S6–9). Notably, these targeted genes were involved in different regulatory networks. In the KEGG analysis, these targeted genes of miR-28-5p, miR-15a and miR-16, miR-660 respectively involved in 77, 70, 83 and 33 different metabolic networks with oncogenic potential (Table S7–10).

## Discussion

In this study, we uncover high *HIP1* expression could predict unfavorable overall survival in AML patients. Additionally, we found distinct microRNA signatures associated with high *HIP1* expression in AML. These results were also validated in an independent cohort of AML patients. Thus, we provide sound evidence that *HIP1* expression analysis can add to risk classification and therapy decision making for AML patients.

The *HIP1* gene is located on chromosome 7q11.23 and encodes a 116-kDa protein^6^. This protein can interact with clathrin, actin, and inositol lipid and involve in receptor trafficking, including regulating cell surface expression of receptor tyrosine kinases^6^. The activated tyrosine kinase signal is important for leukemogenesis. Frequent mutations of tyrosine kinase genes like *FLT3, KIT, NRAS* and *JAK2* in de novo AML were well documented. In our study, we found *HIP1* expressers had a higher frequency of *FLT3*-ITD positive in our cohort, although the difference is not significant. By contrast, in patients from the TCGA cohort, high *HIP1* expression was positively correlated with *FLT3*-ITD positive. The discrepancy might be attributed to the lower frequency of *FLT3*-ITD in our cohort than in the Western cohort. We also found patients with high expression of *HIP1* had lower levels of hemoglobin. One possible reason may be the hypothesis that overexpression of *HIP1* in blasts can stabilize or even increase levels of transferrin receptor as reported^9^ and in turn promote utilization of iron for blasts, leading to iron deficiency in normal red blood cells. Interestingly, *HIP1* overexpression enables prostate cancer cells to metastasis through increasing the expression of integrin^6^. The similarities of high *HIP1* expression between FAB subtype M4/M5 blasts and the prostate cancer cells suggest an analogous promoting metastasis role for *HIP1* through regulations of cytoskeletal processes and integrin expression. Analogously, *HIP1* expression might facilitate M4/M5 blasts to migrate into extramedullary organs. What is very interesting and consistent with our results is that Roel G. W. *et al*. show *NPM1* mutant AML blasts have higher level of *HIP1* expression by gene microarray analysis^16^. It is conceivable that high *HIP1* expressers will associate with *DNMT3A* mutations. The reason might be that both *DNMT3A* mutations and *HIP1* overexpression are predominant in FAB M4/M5 subtype patients. However, the reason why *HIP1* expression is negatively associated with *CEBPA* double allele mutation in our cohort but not in TCGA cohort is unclear. As mentioned above, these results implied AML cases with high *HIP1* expression might be more resistant to chemotherapy, and associated with a poorer outcome. In this study, we find that high *HIP1* expressers harbored poor overall survival in two different cohorts. This is consistent with the report that *HIP1* overexpression with oncogenic property is an independent predictor of relapse in patients with prostate cancer^17^. By contrast, Hsu *et al*. reported HIP1 functions as a potential tumor suppressor^18^. They observed that reduced expression of *HIP1* in lung adenocarcinoma cells leads to development of late metastases and poor prognosis. Taken together, these conflicting data in solid tumors indicate that functions of *HIP1* need much more experimental clarification.

In order to further understand the biologic insight of aberrant *HIP1* expression, we conducted the miRNAs analysis in AMLs. Among differentially expressed miRNAs, we found 31 miRNAs had dysregulated expression in our patients. Among the 18 upregulated expression of miRNAs, 4 miRNAs including miR-15a, miR-16-2, miR-28 and miR-660 were validated in a large cohort of patients. More importantly, these 4 miRNAs were also downregulated after silencing *HIP1* expression in both Kasumi-1 and THP-1 cell lines. These results implied that one of the main mechanisms of *HIP1* in the oncogenic propensity might directively or indirectively act through these miRNAs. In order to understand the biological insight of these miRNAs, miRNA-mRNA interaction were carried out in silico analyses. Consequently, these miRNAs could affect 342 out of 1137 (30%) genes that significantly changed between high and low *HIP1* expressers in TCGA data set. These targeted genes were involving in 263 metabolic pathways in KEGG pathway analysis. The miRNAs were functionly involved in several important pathways. For example, *CCND3* gene regulated by miR-28-5p involved in P53 pathway, Wnt signaling pathway, cell cycle and Jak-STAT signaling pathway (Table S7), several targeted genes (*ZYX, VCL, PDPK1, MAPK9, COL1A1,* Tables S8 and 9) of miR-15/16 were involved in adhesion or migration processes; *LFNG* in notch signaling pathway was regulated by miR-660 (Table S10), etc. Moreover, these miRNAs have been proved to be important prognostic markers and novel targets for therapy in cancers. Although miR-15 and miR-16 are mainly reported to be tumor suppressors, they have been reported to be upregulated in various kinds of cancer and be correlated with tumor cells metastasis, indicating their potential roles as oncomiRs^5^. miR-660 expression was used as a good candidate for prognosis prediction in breast cancer^19^. In addition, increased miR-28 expression leads to autonomous growth of hematopoietic cells by constitutive activation of STAT5^20^. These differentially expressed microRNAs may help us further understand the biologic insights of poor survival in patients with high *HIP1* expression.

There are still some limitations in this study. Firstly, we only examine genes of *FLT3*-ITD, *NPM1, CEBPA* and *DNMT3A* mutations, thus we could not exclude other genes like *IDH1/2, TET2, ASXL1* mutations those will confound the prognostic value of *HIP1* expression in AML patients. Secondly, the putative interaction of miRNA and mRNA uncover several important regulatory networks, but luciferase reporter assays are required to further study in the future. Finally, functional study is limited to the silencing *HIP1* expression on proliferation in leukemia cell lines *in vitro*, enforced expression of *HIP1* and *in vivo* models are also required to investigate the oncogenesis of *HIP1*. Therefore, caution in application of our findings is still warranted.

In conclusion, we present high *HIP1* expression as a reliable and powerful prognostic factor for AML.

## Materials and Methods

### Patients

Clinical data were abstracted from medical records of AML patients in Zhejiang Institute of Hematology (ZIH), China. Between January 2010 and July 2015, 270 patients with detailed diagnoses and treatment information were included in this study. WHO classification, conventional cytogenetic banding assay, and molecular analyses were performed as previously described in AML diagnosis^21^. Cytogenetic groups of patients were classified as favorable, intermediate, and unfavorable risk according to the NCCN guideline^22^. Favorable subgroups included t(8; 21)/*AML1-ETO* and inv16/*CBFβ-MYH11*; adverse consisted of t(9; 22), inv(3)/t(3; 3), −5, −7, del(5q), del(7p), 11q23 and complex translocations; intermediate subgroups contained cytogenetically normal (CN) and AML with other cytogenetic abnormalities. CN -AML was defined as AML with the karyotype 46, XY [20] or 46 XX [20] in all 20 metaphase cells analyzed. Patients were treated with intensive induction chemotherapy as previous reported^23^,^24^. In the consolidation therapy, younger patients were treated with a high-dose cytarabine-based chemotherapy^23^. The chemotherapy consolidation for elderly patients was decided by the physicians in an individualized manner, as described previously^23^. No patient in our study received allogeneic transplantation. Patients with secondary AML or acute promyelocytic leukemia were excluded. This study was approved by the Research Ethics Committee of the First Affiliated Hospital, College of Medicine, Zhejiang University (No. 2016313). Written informed consent was obtained from all participating subjects. All the study methods were carried out in accordance with the approved guidelines.

### Cytogenetic and Gene mutation analysis

The bone marrow (BM) samples of de novo AML patients were analyzed by R-banding analysis. Chromosomal abnormalities were described according to the International System for Human Cytogenetic Nomenclature^25^. DNA and RNA samples of AML patients were obtained from mononuclear cells isolated by Ficoll gradient centrifugation from bone marrow samples at primary diagnosis. Gene mutations of *NPM1, FLT3*-ITD, and *CEBPA* were analyzed by whole-gene sequencing as previously described^26^. RNA samples were used to determine *PML-RARA, AML1-ETO*, and *CBFβ-MYH11* fusion genes by reverse transcription polymerase chain reaction (RT-PCR).

### Quantitative reverse transcriptase-PCR

RNA was extracted using RNeasy Mini kit (Qiagen, Venlo, Netherlands) and first-strand complementary DNA synthesis was performed using the MMLV systems (Life Technologies). Quantitative PCR was performed in triplicate using SYBR-Green PCR Master Mix kit (Takara, Japan) on an IQ5 real time PCR instrument (Bio-Rad, USA), using standard settings: 95 °C (1 min), 40 cycles of 95 °C (5 s) and 60 °C (1 min). mRNA levels were normalized to GAPDH housekeeping gene. The following primers were used for quantitative PCR: *HIP1* 5′-GCGGCTCATTCAGATCCCC-3′ (sense) and 5′-GAGGTCATCCTTCTCTAGGACTG-3′ (antisense*); GAPDH* (control), 5′-ATGGGGAAGGTGAAGGTCG-3′ (sense) and 5′-GGGTCATTGATGGCAACAATATC-3′ (antisense). PCR reactions were performed in a total volume of 25 μl containing of 1 μl of 100 ng/μl sample cDNA, 12.5 μl of 2 × PCR Mix, 1 μl of 0.5 μM of each primer, and 10.5 μl of ddH2O.

### MicroRNA experiments

For the miRNA profiling, total RNA was extracted and purified using mirVana™ miRNA Isolation Kit (Ambion, Austin, TX, US) following the manufacturer’s instructions. RNA integrity number (RIN) was assessed by an Agilent Bioanalyzer 2100 (Agilent technologies, Santa Clara, CA, US). miRNA expression was performed using the Agilent Human miRNA Microarray Kit Version 16.0. Total RNA (100 ng) was hybridized per sample and processed according to the manufacturer’s instructions. The arrays were scanned by an Agilent Technology G2565BA scanner. The scanned images were gridded and analyzed with Agilent Feature Extraction Software Version 10.7. Raw data were normalized by quantile algorithm using Gene Spring Software 11.0. Each microRNA signature was represented by the average of its expression value of replicate probes.

### Cell culture and transfections

Kasumi-1 and THP-1 cell lines were purchased from a typical cell culture collection Committee of the Chinese Academy of Sciences Library. Cells were cultured in RPMI 1640 medium (Corning, USA) containing 10% fetal bovine serum. Cells were maintained at 37 °C, 5% CO2, 95% air and 100% relative humidity. The *HIP1* siRNA and negative control were purchased from Vigene Biosciences (Shangdong, China). *HIP1* siRNA and negative control were transfected in THP-1 and Kasumi-1 cells using protocol provided by the manufacturer. To monitor the effect of siRNA on gene silencing, 5 × 10^5^ cells were seeded in 6-well plates in 2 mL medium containing 5 μl LipofectamineTM3000 transfection reagent and 50 nM siRNA for 72 hours. Down-regulation of *HIP1* expression was detected by quantitative real-time PCR analysis. The oligo sequences are available in the supplementary methods.

### Cell proliferation assay

Cell proliferation assays were performed in triplicate with THP-1 and Kasumi-1 cells transfected with and without the HIP1-siRNA and negative control (NC) siRNA. The experiment was subdivided into four groups: HIP1 siRNA, NC siRNA, blank control and culture only. The treated cells with only lipofectamine reagent were considered as a blank control. Specifically, 100 ul of cells (5 × 10^5^ cells/ml) were plated into 96-well plates. 10 ul of CellTiter 96 Aqueous One Solution Cell Proliferation Assay solution (Promega, USA) was added to each well at post transfection 24, 48 and 72 hours. Plates were read in 490 nm. Growth curves were generated by quantifying the relative number of viable cells.

### Expression of microRNAs after silencing *HIP1* expression

After 72 hours, total RNA was isolated from transfected cells. qTR-PCR was performed using ALL-in-one miRNA real-time quantitative reverse transcription PCR (qRT-PCR) detection kit (GeneCopeia,USA). Total RNA (2 μg) was incubated with miRNA reverse transcription (RT) reagents at 37 °C for 60 min, 85 °C 5 mim in a total volume of 25 μl. The cDNA product was stored −20 °C until being analyzed with RT-PCR. To detect the miRNAs, 2 μl cDNA product was amplified using 2 μl miRNA qPCR primer (2 μM), 2 μl the universal adaptor PCR primer (2 μM) and 10 μl ALL-in-one PCR mix in 20 μl PCR System. The reactions were incubated in a 96-well plate at 95 °C for 10 min, followed by 40 cycles of 95 °C for 10 sec, 58 °C for 20 sec and 72 °C for 10 sec. All sample were run in triplicate. The relative quantification of the target gene expression was determined using the 2^−ΔΔCT^ method and U6 was used for normalization. All primers were seen in the Supplementary Methods.

### Definition of clinical end points and statistical analysis

Patient characteristics were summarized using descriptive statistics, which included frequency counts, median, and range. The main objective of this study was to evaluate the prognostic impacts of *HIP1* expression on Overall survival (OS) of AML patients. OS was defined as time from date of diagnosis until death due to any cause or the last follow-up. We used AML cohort from TCGA (https://tcga-data.nci.nih.gov/tcga/) as a validation cohort, which contains publicly available data of gene microarray expression and clinical information. Determination of optimal cutoff value for *HIP1* expression in our study and the validation TCGA cohort was done with Cutoff Finder using log-rank test (http://molpath.charite.de/cutoff/). To test the robustness of HIP1 gene as a prognostic biomarker, the resampling statistics of the multiple survival screening (MSS) algorithm was used as reported by Jie Li *et al*.^14^. First, we used the TCGA dataset to generate the survival genes with the “samr” R package^27^. Second, we generated 36 random datasets (RDSs) with the 44 of 131 high HIP1 expressers and 22 of 66 low HIP1 expressers in the TCGA cohort. Additionally, we generate 10000 random gene sets (RGSs) each containing 30 genes which were randomly selected from the 45 survival genes. For each RGS-RDS pair, we used the R-code of MSS as reported by Jie Li *et al*.^14^ to calculate the survival screening P-value of the RGS and identify the top 30 genes signatures. The proportional-hazards assumption was checked for each variable before fitting Cox models. Variables with a p-value < 0.2 and the well-established predictors were selected as adjustment covariates into the multivariable analyses. A nonparameter T-test was used to test for the difference of microRNA signatures between high and low *HIP1* expressers. Hierarchical clustering based on expression levels of these microRNAs was performed and visualized by heatmap. Interaction of miRNA and mRNA integrative analyses in silico were using the mirtar platform (http://mirtar.mbc.nctu.edu.tw/human/index.php). All statistical analyses were conducted with R statistic packages, version 2.15.0 (www.r-project.org). The two-sided level of significance was set at p-value < 0.05.

## Additional Information

**How to cite this article:** Wang, J. *et al*. Prognostic significance of huntingtin interacting protein 1 expression on patients with acute myeloid leukemia. *Sci. Rep.* **7**, 45960; doi: 10.1038/srep45960 (2017).

**Publisher's note:** Springer Nature remains neutral with regard to jurisdictional claims in published maps and institutional affiliations.

## Supplementary Material

Supplementary Information

## Figures and Tables

**Table 1:** Characteristics of AML patients by high and low *HIP1* expression.

Variables	Low expression	High expression	P value
Number, (%)	90 (33)	180 (67)	
Age, median(range), years	42 (16,82)	50 (15,82)	0.013
Male, n(%)	48 (53)	108 (60)	0.360
WBC, median(range), ×10^^9^/L^1^	20.9 (0.6,354)	26.2 (0.2,293)	0.655
HB, median(range), g/L^2^	88 (35,136)	79 (34,141)	0.026
PLT, median(range), ×10^^9^/L^3^	36 (6,778)	45 (2,776)	0.132
BM blast, median(range), %^4^	73 (21,97)	68 (15,98)	0.210
FAB classification, n(%)^5^			0.025
M0	8 (9)	13 (7)	
M1	14 (16)	14 (8)	
M2	46 (51)	74 (41)	
M4	3 (3)	21 (12)	
M5	16 (18)	50 (28)	
M6	3 (3)	4 (2)	
Unclassified	0 (0)	4 (2)	
Karyotype risk, n(%)			0.037
Favorable	8 (9)	4 (2)	
Intermediate	75 (83)	155 (86)	
Unfavorable	7 (8)	21 (12)	
Genes mutations, n(%)			
*FLT3*-ITD	13 (14)	38 (22)	0.216
*NPM1*	15 (17)	52 (30)	0.036
*CEBPA*^*DM6*^	22 (24)	13 (7)	<0.001
*DNMT3A*	4 (5)	22 (13)	0.031
Treatment^7^			0.135
IA	65 (72)	112 (62)	
DA	25 (28)	68 (38)	

Abbreviations: ^1^WBC, white blood cell; ^2^HB, hemoglobin; ^3^PLT, platelet counts; ^4^BM, bone marrow; ^5^FAB, French–American–British classification systems; ^6^DM: Double-allele. ^7^The protocols used for induction therapy in different groups including idarubicin/Ara-C (IA)-based treatment group and donorubicin/Ara-C (DA)-based treatment group.

**Table 2:** Multivariable analysis for overall survival in AML patients from ZIH cohort.

Variables	HR (95%CI)	P value
*HIP1* expression (High vs. Low)	1.658 (1.068,2.576)	0.024
Age	1.022 (1.010,1.035)	<0.001
WBC^1^	1.005 (1.003,1.008)	<0.001
Karyotype		
Intermediate vs. favorable	2.03 (0.633,6.507)	0.234
Poor vs. favorable	4.501 (1.306,15.514)	0.017
Gene mutations (mutation vs. wild-type)		
*FLT3*-ITD	1.567 (1.007,2.440)	0.047
*NPM1*	0.606 (0.381,0.964)	0.034
*CEBPA*^*DM2*^	0.489 (0.250,0.957)	0.037
*DNMT3A*	1.86 (1.062,3.258)	0.030
Treatment^3^		
IA vs. DA	0.964 (0.660,1.408)	0.850

Abbreviations: ^1^WBC,white blood cell; ^2^DM: Double-allele. ^3^The protocols used for induction therapy in different groups including donorubicin/Ara-C (DA)-based treatment group and idarubicin/Ara-C (IA)-based; CI, confidence intervals; HR, hazard ratio. Age and WBC are taken as continuous variables.
