# Supplementary material for: Prognostic significance of huntingtin interacting protein 1 expression on patients with acute myeloid leukemia
Source: Sci Rep. 2017 Apr 28;7:45960. doi: 10.1038/srep45960 (PMC5408226; doi:10.1038/srep45960)
Supplement: Supplementary Information [file srep45960-s1.pdf]

Prognostic significance of huntingtin interacting protein 1 expression on patients with acute myeloid leukemia

Authors:

Jinghan Wang1,2,3*, Mengxia Yu2*, Qi Guo 4, Qiuling Ma 5, Chao Hu2; Zhi Xin Ma2, Xiufeng Yin2, Xia Li2, Yungui Wang2,3, Hanzhang Pan2,3, Dongmei Wang2,3, Jiansong Huang2,3, Haitao Meng1,2,3, Hongyan Tong1,2,3,Wenbin Qian1,2,3, Jie Jin1,2,3※

Affiliations:

1 Department of Hematology, The First Affiliated Hospital, Zhejiang University College of Medicine, Hangzhou, China.

2 Institute of Hematology, Zhejiang University, Hangzhou, China.

3 Key Laboratory of Hematologic Malignancies, Diagnosis and Treatment, Zhejiang, Hangzhou, China.

4 Department of Nephrology, The First Affiliated Hospital, Zhejiang University, Hangzhou, China

5 Department of Hematology, The Second Affiliated Hospital of Henan University of Traditional Chinese Medicine, Zhengzhou, China

*These authors contributed equally to this work.

※Corresponding author Address: The First Affiliated Hospital, College of Medicine, Zhejiang University, 79# Qing chun Road, Hangzhou, 310003, P.R. China.

Fax: +86 571 87236702

siRNA oligonucleotides

The following siRNA oligonucleotides were used for this study.

HIP1 siRNAs

Hs-HIP1-si-RNA: 5- GCCUCAUGCAAGCUAUUCA-3

3-UGAAUAGCUUGCAUGAGGC-5

Negative control

5-UUCUCCGAACGUGUCACGU-3

3-ACGUGACACGUUCGGAGAA-5

All primers were as followings:

miR-15A: TAGCAGCACATAATGGTTTGTG,

miR-16： TAGCAGCACGTAAATATTGGCG,

miR-28: AAGGAGCTCACAGTCTATTGAG,

miR-660: TACCCATTGCATATCGGAGTTG,

U6: TTCGTGAAGCGTTCCATATTTT.

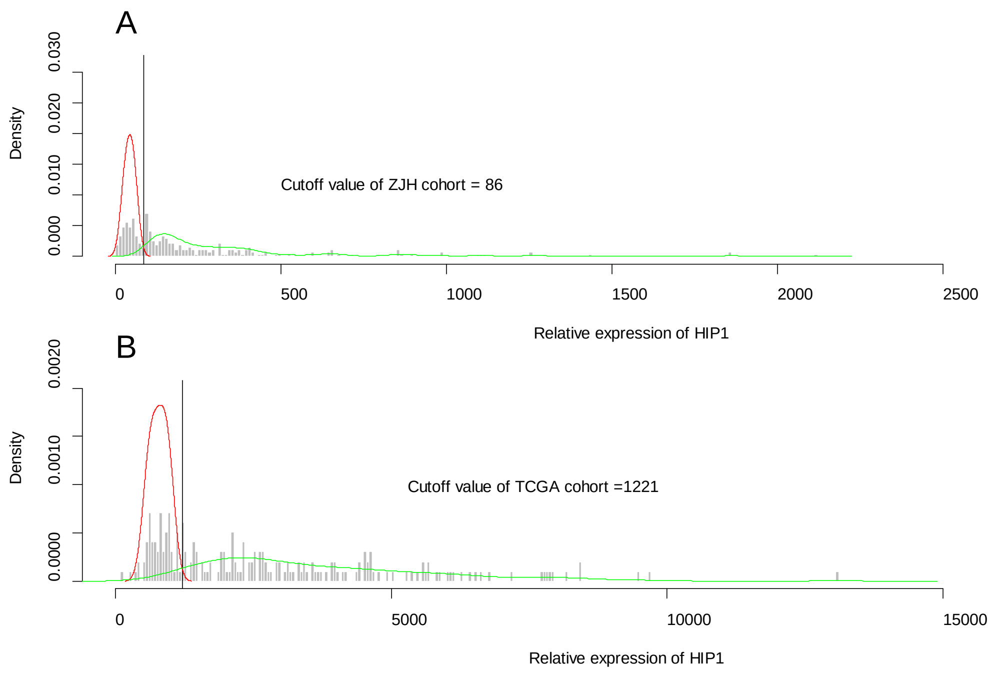

Figure S1 Distribution of *HIP1* expression in ZIH cohort (A) and TCGA cohort (B). The distribution of *HIP1* expression was binormal and exhibited two clusters low and high expressers. Red lines fit the distribution of low expression of *HIP1* and green lines high expression. The value of *HIP1* expression is detected by q RT-PCR in ZJH cohort and RNA sequencing in TCGA cohort. In ZIH cohort, 90 (33%) were classified as low and 180 (67%) high *HIP1* expression. In TCGA cohort, 66 (33%) were defined as low *HIP1* expressers and 131 (67%) as high expressers. Each cutoff value was estimated using Cutoff Finder software analysis, respectively. Relative expression of *HIP1* in the ZJH cohort is multiplied by 100.

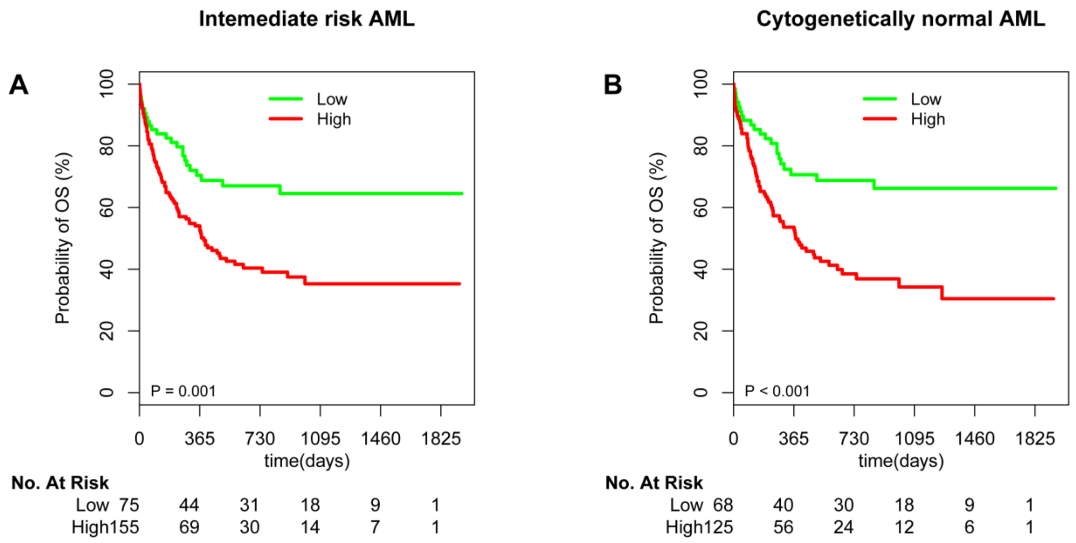

Figure S2. Kaplan-Meier estimates of OS by high and low *HIP1* expression for patients with intermediate risk (A) and cytogenetically normal (B) AML, respectively.

Strata	P value	Low	High	Pi value	Harzard Ratio (95%CI)
HIP1(UV)	<0.001	90	180		
Treatment	<0.001	90	180	0.392	
IA		65	112		
DA		25	68		
Age	<0.001	90	180	0.861	
> 60 yrs		20	58		
<60 yrs		70	122		
Sex	<0.001	90	180	0.839	
Female		42	72		
Male		48	108		
WBC	<0.001	90	180	0.287	
Below mean		59	119		
Above mean		31	61		
HB	<0.001	90	180	0.872	
Below mean		37	104		
Above mean		53	76		
PLT	<0.001	90	180	0.276	
Below mean		67	124		
Above mean		23	56		
Blast%	<0.001	90	180	0.402	
Below mean		38	80		
Above mean		52	100		
FAB	<0.001	90	180	0.844	
Non-M4 or M5		71	109		
M4 or M5		19	71		
Karytope	<0.001	90	180	0.067	
Favorable		8	4		
Intermediate		75	155		
Unfavorable		7	21		
NPM1	<0.001	90	180	0.653	
Wildtype		74	124		
Mutant		15	52		
FLT3ITD	<0.001	90	180	0.354	
Wildtype		77	138		
Mutant		13	38		
CEBPA	<0.001	90	180	0.413	
Others		68	164		
Mutant DM		22	13		
DNMT3A	<0.001	90	180	0.099	
Wildtype		84	145		
Mutant		4	22		

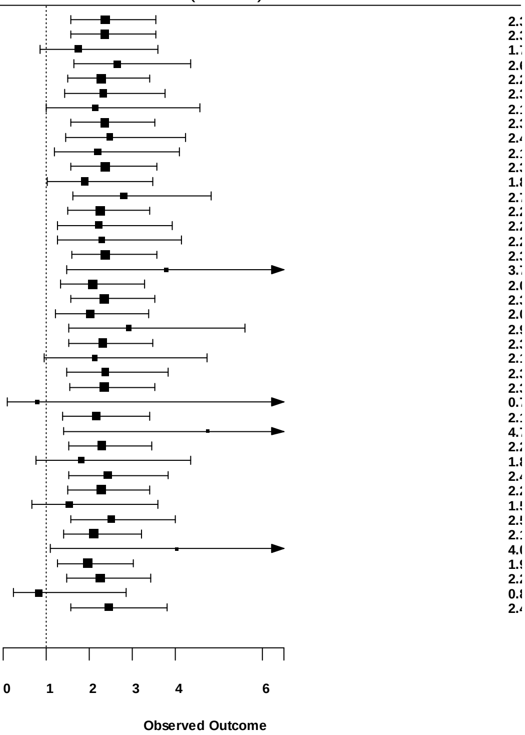

Figure S3. Hazard ratios (HR) of altered *HIP1* mRNA on overall survival by stratified analyses. “P value” represents the p-values after adjustment analyses for each factor, and “Pi value” represents the p-values of interaction between altered levels of *HIP1* mRNA expression and each factor. The prognostic value of *HIP1* expression is significant in univariate analysis (UV) for OS. The adjustment p-values do not strikingly change after adjustment for each factor, implying each adjusted factor does not significantly alter the interpretation of the prognostic value of *HIP1* mRNA expression. The p-values of interaction (Pi value) between *HIP1* mRNA expression and each factor are more than 0.05, indicating no apparent interactive factors exist.

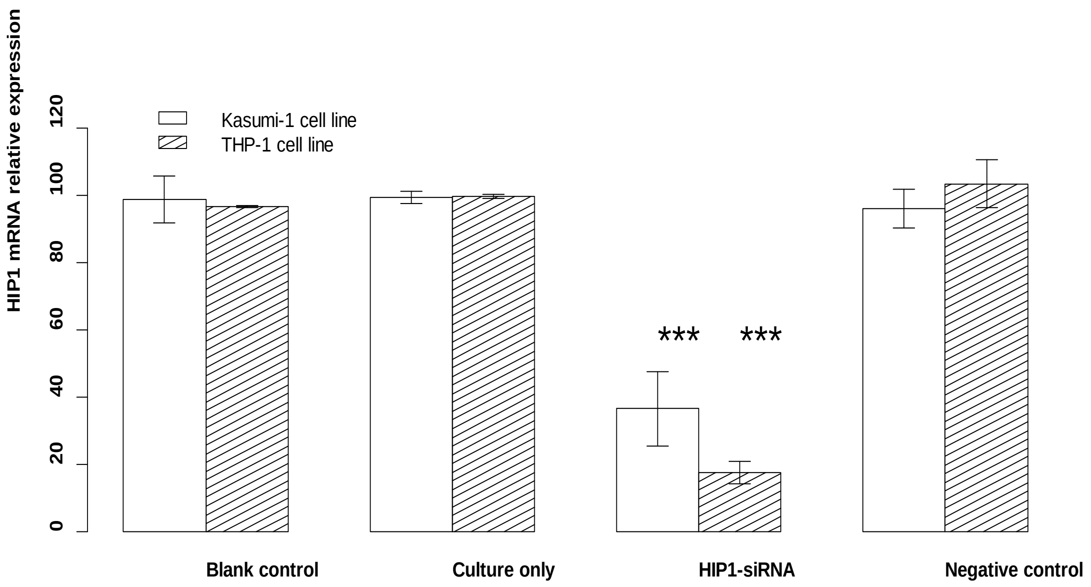

Figure S4. Silencing *HIP1* expression in Kasumi-1 and THP-1 cell lines, respectively. The cells were treated with 50 nM HIP1-siRNA, negative control siRNA, lipofectamine reagent without siRNA (blank control) and culture only. The relative expression of *HIP1* was determined by Real-time PCR analysis after 72 hours transfection. The relative quantification of the HIP1 expression was determined using the $2^{-\Delta\Delta Ct}$ method and GAPDH was used for normalization. Expression levels were analyzed relative to the sample cultured only as 100%. Statistical analysis was performed using one-way ANOVA followed by Tukey post-test. There was significantly decreased expression of *HIP1* in HIP-1-siRNA group compared to negative control. However, no significant changes were seen among negative control, blank control and culture only group. “***” represents P<0.001.

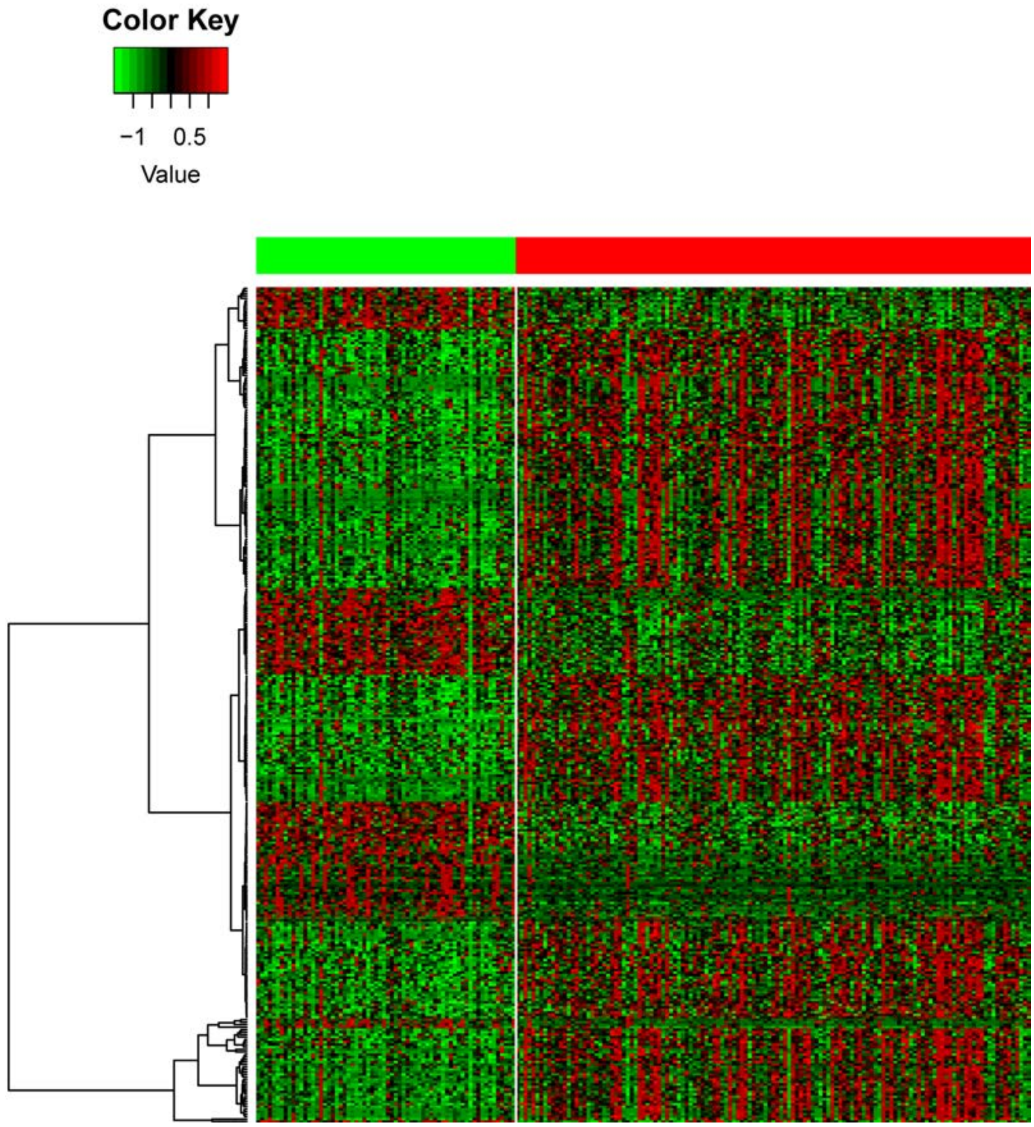

Figure S5. Gene expression profiling of patients with high (red bar) and low (green bar) HIP1 expression in the TCGA cohort.

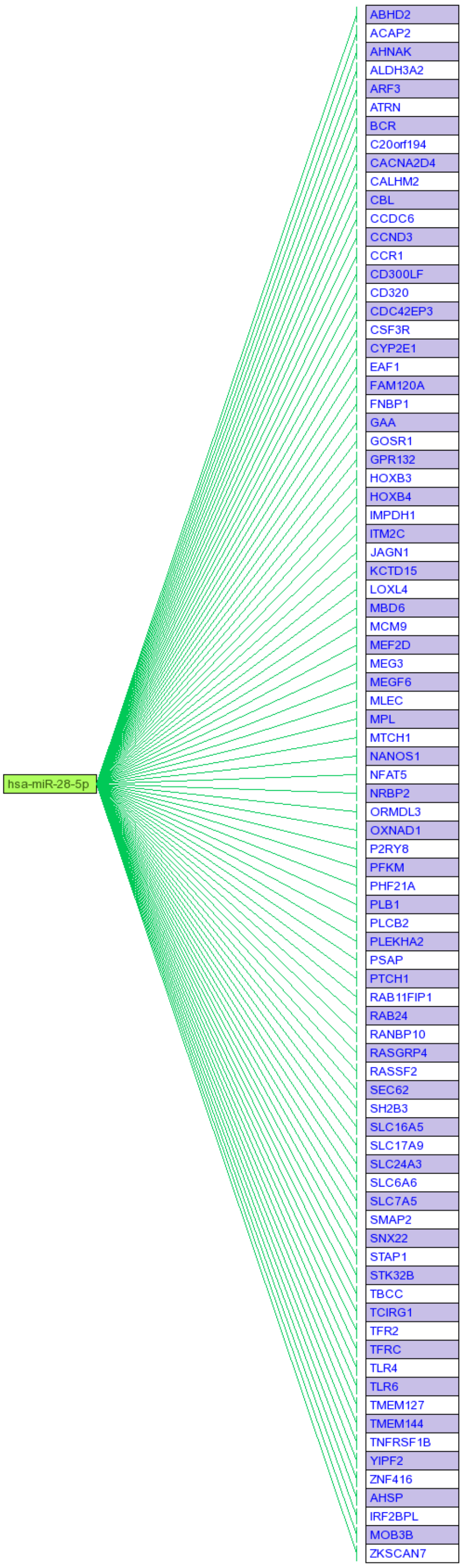

Figure S6. Predicted targeted genes of miR-28-5p.

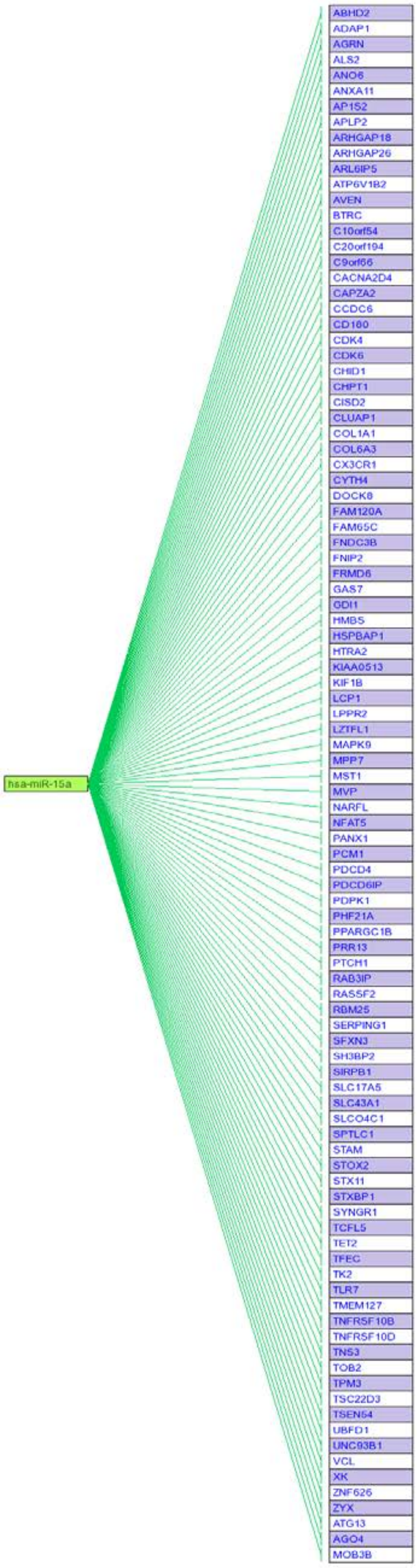

Figure S7. Predicted targeted genes of miR-15a.

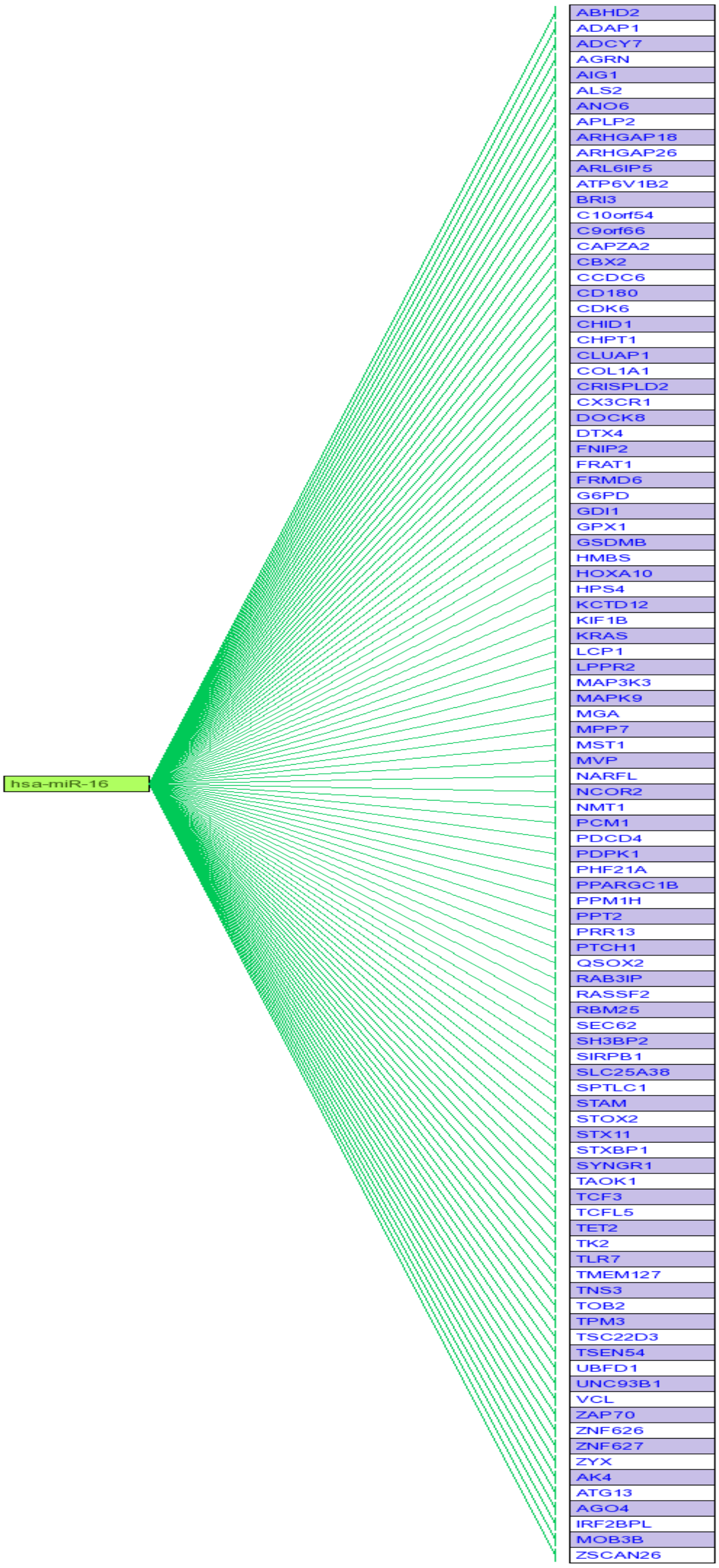

Figure S8. Predicted targeted genes of miR-16.

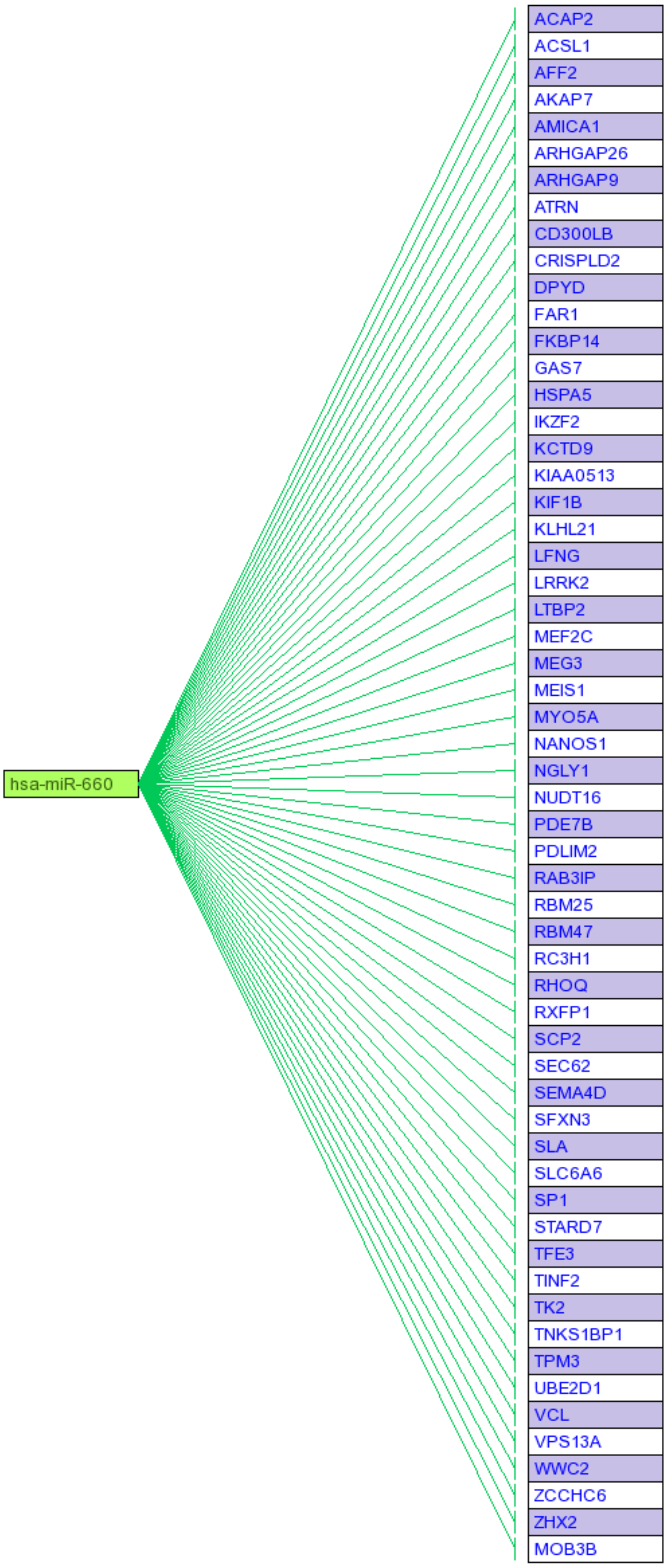

Figure S9. Predicted targeted genes of miR-660.

Table S1. Multivariable analysis for overall survival in AML patients with survival more than 30 days from ZIH cohort.

Variables	HR (95%CI)	P value
HIP1 expression (High vs. Low)	1.766(1.074,2.905)	0.025
Age	1.023(1.009,1.038)	0.001
WBC1	1.003(0.9997,1.006)	0.073
Karyotype		
Intermediate vs. favorable	2.688(0.649,11.136)	0.173
Poor vs. favorable	6.603(1.489,29.288)	0.013
Gene mutations		
FLT3-ITD	1.778(1.067,2.961)	0.027
NPM1	0.489(0.283,0.845)	0.010
CEBPA[DM2]	0.449(0.212,0.954)	0.037
DNMT3A	1.451(0.692,3.042)	0.324
Treatment[3]		
IA vs. DA	0.788(0.506,1.228)	0.293

Abbreviations: [1]WBC, white blood cell; [2]DM: Double-allele. [3]The protocols used for induction therapy in different groups including donorubicin/Ara-C (DA)-based treatment group and idarubicin/Ara-C (IA)-based; CI, confidence intervals; HR, hazard ratio.

Table S2. Univariate and multivariate analyses for complete remission rate in AML patients

Variables	Univariate analysis		Multivariate analysis	
	HR (95%CI)	P value	HR (95%CI)	P value
HIP1 expression(high vs.low)	0. 491(0. 256, 0. 903)	0. 026	0. 617(0. 29, 1. 263)	0. 196
Age	0. 617(0. 290, 1. 263)	0. 196	0. 974(0. 954, 0. 994)	0. 012
WBC[1]	0. 996(0. 993, 1. 000)	0. 066	0. 995(0. 99, 0. 999)	0. 016
Karyotype				
Intermediate vs. favorable	0. 607(0. 092, 2. 389)	0. 527	0. 956(0. 129, 4. 700)	0. 959
Poor vs. favorable	0. 267(0. 036, 1. 256)	0. 126	0. 484(0. 056, 2. 985)	0. 458
Gene mutations	0. 585(0. 307, 1. 140)	0. 108	0. 536(0. 25, 1. 162)	0. 109
FLT3-ITD				
NPM1	0. 794(0. 434, 1. 487)	0. 461	2. 142(0. 989, 4. 884)	0. 06
CEBPA^{DM2}	3. 081(1. 163, 10. 657)	0. 041	2. 665(0. 903, 9. 994)	0. 102
DNMT3A	0. 421(0. 183, 0. 991)	0. 042	0. 492(0. 187, 1. 305)	0. 148
Treatment[3](IA vs. DA)	0. 390(0. 223, 0. 681)	0. 001	0. 494(0. 262, 0. 932)	0. 029

Abbreviations: [1]WBC, white blood cell; [2]DM: Double-allele.[3]Treatment protocols used for induction therapy in different groups including donorubicin/Ara-C (DA)-based treatment group and idarubicin/Ara-C (IA)-based; CI, confidence intervals; HR, hazard ratio.

Table S3. Characteristics of AML patients by high and low *HIP1* expression from the TCGA cohort

Variables	Low expression	High expression	P value
Number,(%)	66(33)	131(67)	
Age, median(range),years	53.5(18,82)	58(21,88)	0.04
Male, n(%)	38(58)	68(52)	0.547
WBC, median(range),×10^9/L[1]	5.6(0.4,111)	30.8(0.8,298.4)	<0.001
Percent BM blast, median(range),%[4]	68.5(30,100)	75(30,100)	0.283
FAB classification, n(%)[5]			<0.001
M0	4(6)	13(10)	
M1	15(23)	31(24)	
M2	20(30)	24(18)	
M3	17(26)	2(2)	
M4	3(5)	38(29)	
M5	3(5)	19(15)	
M6	2(3)	1(1)	
M7	2(3)	1(1)	
Unclassified	0(0)	2(2)	
Karyotyperisk,n(%)			<0.001
Favorable	22(33)	14(11)	
Intermediate	27(41)	87(66)	
Unfavorable	14(21)	28(21)	
N.D.	3(5)	2(2)	
Genesmutations,n(%)			
FLT3-ITD	11(17)	44(34)	0.02
NPM1	7(11)	47(36)	<0.001
CEBPA[DM6]	2(3)	3(2)	1

DNMT3A	8(12)	42(32)	0.003
IDH1	4(6)	15(11)	0.309
IDH2	5(8)	14(11)	0.613
Transplantation			1
Non-BMT	37(56)	73(56)	
BMT	29(44)	58(44)	

Abbreviations:[1]WBC,white blood cell; [2]HB, hemoglobin; [3]PLT, platelet counts; [4]BM, bone marrow; [5]FAB, French–American–British classification systems; [6]DM: Double-allele. BMT, bone marrow transplantation.

Table S4 The top 30 frequent genes predictive to survival in multiple survival screening analyses.

ID	Gene symbols
1	NPY2R
2	P2RY8
3	ZNF532
4	RDH10
5	MYB
6	DUSP7
7	WNK3
8	MAOB
9	KIAA0125
10	LOC100272216
11	DET1
12	PLIN3
13	CDO1
14	DCP2
15	KIAA0125
16	LOC91316
17	SPATS2L
18	SPON1
19	KBTBD8
20	CLEC11A
21	VSTM1
22	ELANE
23	GMFB

24	KCNK1
25	PLA2G4A
26	KCTD15
27	PLD1
28	CALR
29	HIP1
30	SLC24A3

Table S5. Univariate and multivariable analysis for overall survival in AML patients from TCGA cohort

	Univariate analysis		Multivariate analysis	
Variables	HR (95%CI)	P value	HR (95%CI)	P value
HIP1 expression(high vs.low)	1.92(1.30,2.84)	0.001	1.558(1.017,2.385)	0.041
Age	1.04(1.027,1.054)	<0.001	1.027(1.011,1.043)	0.001
WBC[1]	1.003(0.999,1.006)	0.122	1.001(0.997,1.005)	0.702
Karyotype				
Intermediate vs. favorable	3.202(1.742,5.883)	<0.001	3.244(1.613,6.524)	0.001
Poor vs. favorable	4.561(2.346,8.865)	<0.001	6.363(3.007,13.466)	<0.001
CytoNDvs .favorable	6.851(2.186,21.468)	0.001	11.242(3.402,37.157)	<0.001
Gene mutations				
FLT3-ITD	1.33(0.91,1.94)	0.146	1.571(0.974,2.533)	0.064
NPM1	1.22(0.84,1.78)	0.303	0.743(0.442,1.25)	0.263
CEBPA[DM2]	0.63(0.2,2.0)	0.436	1.474(0.413,5.265)	0.551
DNMT3A	1.62(1.11,2.36)	0.013	1.188(0.77,1.832)	0.437
IDH1	0.84(0.47,1.53)	0.572	0.907(0.476,1.732)	0.768
IDH2	1.1(0.63,1.93)	0.729	0.721(0.397,1.309)	0.282
BMT vs non-BMT	0.542(0.382,0.771)	0.001	0.445(0.287,0.69)	<0.001

Abbreviations:[1]WBC, white blood cell; [2]DM: Double-allele. CI, confidence intervals; HR, hazard ratio. BMT, bone marrow transplantation.

Table S6. Characteristics of CN-AML patients using for microRNA profiling analysis.

Variables	Low expression	High expression	P value
Number, (%)	5(45)	6(56)	
Age, median(range),years	37(20, 73)	56(20, 78)	0.47
Male, n(%)	38(58)	68(52)	0.547
WBC, median(range),×10^9/L[1]	29(9.7, 259)	29(12, 200)	0.89
Percent BM blast, median(range),%[2]	75(54,91)	79(48,92)	0.86
FAB classification, n(%)			0.40
M0	0(0)	2(33)	
M1	2(40)	0(0)	
M2	1(20)	2(33)	
M4	1(20)	0(0)	
M5	1(20)	2(33)	
Genes mutations, n(%)			
FLT3-ITD	1(20)	1(17)	0.89
NPM1	1(20)	1(17)	0.89
CEBPA^{DM3}	1(20)	0(0)	0.70
DNMT3A	0(0)	3(50)	0.18

Abbreviations: [1]WBC, white blood cell; [2]BM bone marrow; [3]DM: Double-allele.

Table S7 Targeted genes and KEGG pathways of hsa-miR-28-5p

1	Endocytosis - Homo sapiens (human)[hsa04144]
	TFRC, SMAP2, RAB11FIP1, CBL, ACAP2,
2	Jak-STAT signaling pathway - Homo sapiens (human)[hsa04630]
	MPL, CSF3R, CCND3, CBL,
3	Galactose metabolism - Homo sapiens (human)[hsa00052]
	PFKM, GAA,
4	Lysosome - Homo sapiens (human)[hsa04142]
	TCIRG1, PSAP, GAA,
5	Glycolysis / Gluconeogenesis - Homo sapiens (human)[hsa00010]
	PFKM, ALDH3A2,
6	Limonene and pinene degradation - Homo sapiens (human)[hsa00903]
	ALDH3A2,
7	Chronic myeloid leukemia - Homo sapiens (human)[hsa05220]
	CBL, BCR,
8	Wnt signaling pathway - Homo sapiens (human)[hsa04310]
	PLCB2, NFAT5, CCND3,
9	Pathways in cancer - Homo sapiens (human)[hsa05200]
	PTCH1, CSF3R, CCDC6, CBL, BCR,
10	Cytokine-cytokine receptor interaction - Homo sapiens (human)[hsa04060]
	TNFRSF1B, MPL, CSF3R, CCR1,
11	beta-Alanine metabolism - Homo sapiens (human)[hsa00410]
	ALDH3A2,
12	T cell receptor signaling pathway - Homo sapiens (human)[hsa04660]

	NFAT5, CBL,
13	Ascorbate and aldarate metabolism - Homo sapiens (human)[hsa00053]
	ALDH3A2,
14	Protein export - Homo sapiens (human)[hsa03060]
	SEC62,
15	Toll-like receptor signaling pathway - Homo sapiens (human)[hsa04620]
	TLR6, TLR4,
16	Collecting duct acid secretion - Homo sapiens (human)[hsa04966]
	TCIRG1,
17	Histidine metabolism - Homo sapiens (human)[hsa00340]
	ALDH3A2,
18	Pentose phosphate pathway - Homo sapiens (human)[hsa00030]
	PFKM,
19	Butanoate metabolism - Homo sapiens (human)[hsa00650]
	ALDH3A2,
20	Thyroid cancer - Homo sapiens (human)[hsa05216]
	CCDC6,
21	Linoleic acid metabolism - Homo sapiens (human)[hsa00591]
	CYP2E1,
22	Propanoate metabolism - Homo sapiens (human)[hsa00640]
	ALDH3A2,
23	Fructose and mannose metabolism - Homo sapiens (human)[hsa00051]
	PFKM,
24	Pyruvate metabolism - Homo sapiens (human)[hsa00620]

	ALDH3A2,
25	SNARE interactions in vesicular transport - Homo sapiens (human)[hsa04130]
	GOSR1,
26	Vibrio cholerae infection - Homo sapiens (human)[hsa05110]
	TCIRG1,
27	Hedgehog signaling pathway - Homo sapiens (human)[hsa04340]
	PTCH1,
28	Amyotrophic lateral sclerosis (ALS) - Homo sapiens (human)[hsa05014]
	TNFRSF1B,
29	Glycerolipid metabolism - Homo sapiens (human)[hsa00561]
	ALDH3A2,
30	Hematopoietic cell lineage - Homo sapiens (human)[hsa04640]
	TFRC, CSF3R,
31	Pathogenic Escherichia coli infection - Homo sapiens (human)[hsa05130]
	TLR4,
32	Basal cell carcinoma - Homo sapiens (human)[hsa05217]
	PTCH1,
33	Drug metabolism - other enzymes - Homo sapiens (human)[hsa00983]
	IMPDH1,
34	Chemokine signaling pathway - Homo sapiens (human)[hsa04062]
	PLCB2, CCR1,
35	Starch and sucrose metabolism - Homo sapiens (human)[hsa00500]
	GAA,

36	Tryptophan metabolism - Homo sapiens (human)[hsa00380]
	ALDH3A2,
37	Epithelial cell signaling in Helicobacter pylori infection - Homo sapiens (human)[hsa05120]
	TCIRG1,
38	Long-term potentiation - Homo sapiens (human)[hsa04720]
	PLCB2,
39	Inositol phosphate metabolism - Homo sapiens (human)[hsa00562]
	PLCB2,
40	Taste transduction - Homo sapiens (human)[hsa04742]
	PLCB2,
41	Adipocytokine signaling pathway - Homo sapiens (human)[hsa04920]
	TNFRSF1B,
42	p53 signaling pathway - Homo sapiens (human)[hsa04115]
	CCND3,
43	Valine, leucine and isoleucine degradation - Homo sapiens (human)[hsa00280]
	ALDH3A2,
44	Arginine and proline metabolism - Homo sapiens (human)[hsa00330]
	ALDH3A2,
45	VEGF signaling pathway - Homo sapiens (human)[hsa04370]
	NFAT5,
46	Leishmania infection - Homo sapiens (human)[hsa05140]
	TLR4,
47	B cell receptor signaling pathway - Homo sapiens (human)[hsa04662]

	NFAT5,
48	Lysine degradation - Homo sapiens (human)[hsa00310]
	ALDH3A2,
49	Hypertrophic cardiomyopathy (HCM) - Homo sapiens (human)[hsa05410]
	CACNA2D4,
50	Cardiac muscle contraction - Homo sapiens (human)[hsa04260]
	CACNA2D4,
51	Dilated cardiomyopathy - Homo sapiens (human)[hsa05414]
	CACNA2D4,
52	ErbB signaling pathway - Homo sapiens (human)[hsa04012]
	CBL,
53	GnRH signaling pathway - Homo sapiens (human)[hsa04912]
	PLCB2,
54	Gap junction - Homo sapiens (human)[hsa04540]
	PLCB2,
55	Melanogenesis - Homo sapiens (human)[hsa04916]
	PLCB2,
56	Arrhythmogenic right ventricular cardiomyopathy (ARVC) - Homo sapiens (human)[hsa05412]
	CACNA2D4,
57	Arachidonic acid metabolism - Homo sapiens (human)[hsa00590]
	CYP2E1,
58	MAPK signaling pathway - Homo sapiens (human)[hsa04010]
	RASGRP4, CACNA2D4,

59	Phosphatidylinositol signaling system - Homo sapiens (human)[hsa04070]
	PLCB2,
60	Long-term depression - Homo sapiens (human)[hsa04730]
	PLCB2,
61	Fatty acid metabolism - Homo sapiens (human)[hsa00071]
	ALDH3A2,
62	Neurotrophin signaling pathway - Homo sapiens (human)[hsa04722]
	SH2B3,
63	Insulin signaling pathway - Homo sapiens (human)[hsa04910]
	CBL,
64	Vascular smooth muscle contraction - Homo sapiens (human)[hsa04270]
	PLCB2,
65	Ubiquitin mediated proteolysis - Homo sapiens (human)[hsa04120]
	CBL,
66	Natural killer cell mediated cytotoxicity - Homo sapiens (human)[hsa04650]
	NFAT5,
67	Axon guidance - Homo sapiens (human)[hsa04360]
	NFAT5,
68	Alzheimer's disease - Homo sapiens (human)[hsa05010]
	PLCB2,
69	Cell cycle - Homo sapiens (human)[hsa04110]
	CCND3,
70	Calcium signaling pathway - Homo sapiens (human)[hsa04020]

	PLCB2,
71	Focal adhesion - Homo sapiens (human)[hsa04510]
	CCND3,
72	Huntington's disease - Homo sapiens (human)[hsa05016]
	PLCB2,
73	Drug metabolism - cytochrome P450 - Homo sapiens (human)[hsa00982]
	CYP2E1,
74	Neuroactive ligand-receptor interaction - Homo sapiens (human)[hsa04080]
	P2RY8,
75	Oxidative phosphorylation - Homo sapiens (human)[hsa00190]
	TCIRG1,
76	Purine metabolism - Homo sapiens (human)[hsa00230]
	IMPDH1,
77	Metabolism of xenobiotics by cytochrome P450 - Homo sapiens (human)[hsa00980]
	CYP2E1,

Table S8. Targeted genes and KEGG pathways of hsa-miR-15a

1	Focal adhesion - Homo sapiens (human)[hsa04510]
	ZYX, VCL, PDPK1, MAPK9, COL6A3, COL1A1,
2	Non-small cell lung cancer - Homo sapiens (human)[hsa05223]
	PDPK1, CDK6, CDK4,
3	p53 signaling pathway - Homo sapiens (human)[hsa04115]
	TNFRSF10B, CDK6, CDK4,
4	Pancreatic cancer - Homo sapiens (human)[hsa05212]
	MAPK9, CDK6, CDK4,
5	Natural killer cell mediated cytotoxicity - Homo sapiens (human)[hsa04650]
	TNFRSF10D, TNFRSF10B, SH3BP2, NFAT5,
6	Thyroid cancer - Homo sapiens (human)[hsa05216]
	TPM3, CCDC6,
7	T cell receptor signaling pathway - Homo sapiens (human)[hsa04660]
	NFAT5, MAPK9, CDK4,
8	Hedgehog signaling pathway - Homo sapiens (human)[hsa04340]
	PTCH1, BTRC,
9	Pathways in cancer - Homo sapiens (human)[hsa05200]
	TPM3, PTCH1, MAPK9, CDK6, CDK4, CCDC6,
10	Epithelial cell signaling in Helicobacter pylori infection - Homo sapiens (human)[hsa05120]
	MAPK9, ATP6V1B2,
11	Melanoma - Homo sapiens (human)[hsa05218]
	CDK6, CDK4,

12	Chronic myeloid leukemia - Homo sapiens (human)[hsa05220]	
	CDK6, CDK4,	
13	Small cell lung cancer - Homo sapiens (human)[hsa05222]	
	CDK6, CDK4,	
14	Wnt signaling pathway - Homo sapiens (human)[hsa04310]	
	NFAT5, MAPK9, BTRC,	
15	Hypertrophic cardiomyopathy (HCM) - Homo sapiens (human)[hsa05410]	
	TPM3, CACNA2D4,	
16	Cardiac muscle contraction - Homo sapiens (human)[hsa04260]	
	TPM3, CACNA2D4,	
17	Dilated cardiomyopathy - Homo sapiens (human)[hsa05414]	
	TPM3, CACNA2D4,	
18	Apoptosis - Homo sapiens (human)[hsa04210]	
	TNFRSF10D, TNFRSF10B,	
19	Collecting duct acid secretion - Homo sapiens (human)[hsa04966]	
	ATP6V1B2,	
20	Toll-like receptor signaling pathway - Homo sapiens (human)[hsa04620]	
	TLR7, MAPK9,	
21	Glioma - Homo sapiens (human)[hsa05214]	
	CDK6, CDK4,	
22	Insulin signaling pathway - Homo sapiens (human)[hsa04910]	
	PDPK1, MAPK9,	

23	Aldosterone-regulated sodium reabsorption - Homo sapiens (human)[hsa04960]
	PDPK1,
24	Porphyrin and chlorophyll metabolism - Homo sapiens (human)[hsa00860]
	HMBS,
25	Bladder cancer - Homo sapiens (human)[hsa05219]
	CDK4,
26	Lysosome - Homo sapiens (human)[hsa04142]
	SLC17A5, AP1S2,
27	Type II diabetes mellitus - Homo sapiens (human)[hsa04930]
	MAPK9,
28	SNARE interactions in vesicular transport - Homo sapiens (human)[hsa04130]
	STX11,
29	mTOR signaling pathway - Homo sapiens (human)[hsa04150]
	PDPK1,
30	Vibrio cholerae infection - Homo sapiens (human)[hsa05110]
	ATP6V1B2,
31	Endometrial cancer - Homo sapiens (human)[hsa05213]
	PDPK1,
32	Amyotrophic lateral sclerosis (ALS) - Homo sapiens (human)[hsa05014]
	ALS2,
33	Cell cycle - Homo sapiens (human)[hsa04110]
	CDK6, CDK4,

34	Ether lipid metabolism - Homo sapiens (human)[hsa00565]
	CHPT1,
35	Basal cell carcinoma - Homo sapiens (human)[hsa05217]
	PTCH1,
36	Sphingolipid metabolism - Homo sapiens (human)[hsa00600]
	SPTLC1,
37	Drug metabolism - other enzymes - Homo sapiens (human)[hsa00983]
	TK2,
38	Cytokine-cytokine receptor interaction - Homo sapiens (human)[hsa04060]
	TNFRSF10D, TNFRSF10B, CX3CR1,
39	Complement and coagulation cascades - Homo sapiens (human)[hsa04610]
	SERPING1,
40	NOD-like receptor signaling pathway - Homo sapiens (human)[hsa04621]
	MAPK9,
41	RIG-I-like receptor signaling pathway - Homo sapiens (human)[hsa04622]
	MAPK9,
42	Colorectal cancer - Homo sapiens (human)[hsa05210]
	MAPK9,
43	Adipocytokine signaling pathway - Homo sapiens (human)[hsa04920]
	MAPK9,

44	VEGF signaling pathway - Homo sapiens (human)[hsa04370]
	NFAT5,
45	B cell receptor signaling pathway - Homo sapiens (human)[hsa04662]
	NFAT5,
46	PPAR signaling pathway - Homo sapiens (human)[hsa03320]
	PDPK1,
47	Fc epsilon RI signaling pathway - Homo sapiens (human)[hsa04664]
	MAPK9,
48	Progesterone-mediated oocyte maturation - Homo sapiens (human)[hsa04914]
	MAPK9,
49	Prostate cancer - Homo sapiens (human)[hsa05215]
	PDPK1,
50	Endocytosis - Homo sapiens (human)[hsa04144]
	STAM, PDCD6IP,
51	ErbB signaling pathway - Homo sapiens (human)[hsa04012]
	MAPK9,
52	GnRH signaling pathway - Homo sapiens (human)[hsa04912]
	MAPK9,
53	Arrhythmogenic right ventricular cardiomyopathy (ARVC) - Homo sapiens (human)[hsa05412]
	CACNA2D4,
54	Glycerophospholipid metabolism - Homo sapiens (human)[hsa00564]

	CHPT1,
55	Adherens junction - Homo sapiens (human)[hsa04520]
	VCL,
56	MAPK signaling pathway - Homo sapiens (human)[hsa04010]
	MAPK9, CACNA2D4,
57	ECM-receptor interaction - Homo sapiens (human)[hsa04512]
	COL6A3, COL1A1, AGRN,
58	Neurotrophin signaling pathway - Homo sapiens (human)[hsa04722]
	MAPK9,
59	Parkinson's disease - Homo sapiens (human)[hsa05012]
	HTRA2,
60	Ubiquitin mediated proteolysis - Homo sapiens (human)[hsa04120]
	BTRC,
61	Spliceosome - Homo sapiens (human)[hsa03040]
	RBM25,
62	Jak-STAT signaling pathway - Homo sapiens (human)[hsa04630]
	STAM,
63	Axon guidance - Homo sapiens (human)[hsa04360]
	NFAT5,
64	Tight junction - Homo sapiens (human)[hsa04530]
	CDK4,
65	Oocyte meiosis - Homo sapiens (human)[hsa04114]
	BTRC,

66	Leukocyte transendothelial migration - Homo sapiens (human)[hsa04670]
	VCL,
67	Chemokine signaling pathway - Homo sapiens (human)[hsa04062]
	CX3CR1,
68	Pyrimidine metabolism - Homo sapiens (human)[hsa00240]
	TK2,
69	Regulation of actin cytoskeleton - Homo sapiens (human)[hsa04810]
	VCL,
70	Oxidative phosphorylation - Homo sapiens (human)[hsa00190]
	ATP6V1B2,

Table S9. Targeted genes and KEGG pathways of hsa-miR-16

1	Thyroid cancer - Homo sapiens (human)[hsa05216]
	TPM3, KRAS, CCDC6,
2	GnRH signaling pathway - Homo sapiens (human)[hsa04912]
	MAPK9, MAP3K3, KRAS, ADCY7,
3	Non-small cell lung cancer - Homo sapiens (human)[hsa05223]
	PDPK1, KRAS, CDK6,
4	Focal adhesion - Homo sapiens (human)[hsa04510]
	ZYX, VCL, PDPK1, MAPK9, COL1A1,
5	Pancreatic cancer - Homo sapiens (human)[hsa05212]
	MAPK9, KRAS, CDK6,
6	Progesterone-mediated oocyte maturation - Homo sapiens (human)[hsa04914]
	MAPK9, KRAS, ADCY7,
7	Aldosterone-regulated sodium reabsorption - Homo sapiens (human)[hsa04960]
	PDPK1, KRAS,
8	T cell receptor signaling pathway - Homo sapiens (human)[hsa04660]
	ZAP70, MAPK9, KRAS,
9	Notch signaling pathway - Homo sapiens (human)[hsa04330]
	NCOR2, DTX4,
10	Endometrial cancer - Homo sapiens (human)[hsa05213]
	PDPK1, KRAS,
11	Amyotrophic lateral sclerosis (ALS) - Homo sapiens (human)[hsa05014]
	GPX1, ALS2,
12	Pathways in cancer - Homo sapiens (human)[hsa05200]
	TPM3, PTCH1, MAPK9, KRAS, CDK6, CCDC6,

13	Neurotrophin signaling pathway - Homo sapiens (human)[hsa04722]
	MAPK9, MAP3K3, KRAS,
14	Insulin signaling pathway - Homo sapiens (human)[hsa04910]
	PDPK1, MAPK9, KRAS,
15	Glutathione metabolism - Homo sapiens (human)[hsa00480]
	GPX1, G6PD,
16	Epithelial cell signaling in Helicobacter pylori infection - Homo sapiens (human)[hsa05120]
	MAPK9, ATP6V1B2,
17	Colorectal cancer - Homo sapiens (human)[hsa05210]
	MAPK9, KRAS,
18	Melanoma - Homo sapiens (human)[hsa05218]
	KRAS, CDK6,
19	Natural killer cell mediated cytotoxicity - Homo sapiens (human)[hsa04650]
	ZAP70, SH3BP2, KRAS,
20	Chronic myeloid leukemia - Homo sapiens (human)[hsa05220]
	KRAS, CDK6,
21	Fc epsilon RI signaling pathway - Homo sapiens (human)[hsa04664]
	MAPK9, KRAS,
22	Prostate cancer - Homo sapiens (human)[hsa05215]
	PDPK1, KRAS,
23	MAPK signaling pathway - Homo sapiens (human)[hsa04010]
	TAOK1, MAPK9, MAP3K3, KRAS,
24	Chemokine signaling pathway - Homo sapiens (human)[hsa04062]
	KRAS, CX3CR1, ADCY7,

25	Dilated cardiomyopathy - Homo sapiens (human)[hsa05414]
	TPM3, ADCY7,
26	ErbB signaling pathway - Homo sapiens (human)[hsa04012]
	MAPK9, KRAS,
27	Gap junction - Homo sapiens (human)[hsa04540]
	KRAS, ADCY7,
28	Protein export - Homo sapiens (human)[hsa03060]
	SEC62,
29	Dorso-ventral axis formation - Homo sapiens (human)[hsa04320]
	KRAS,
30	Collecting duct acid secretion - Homo sapiens (human)[hsa04966]
	ATP6V1B2,
31	Melanogenesis - Homo sapiens (human)[hsa04916]
	KRAS, ADCY7,
32	Toll-like receptor signaling pathway - Homo sapiens (human)[hsa04620]
	TLR7, MAPK9,
33	Pentose phosphate pathway - Homo sapiens (human)[hsa00030]
	G6PD,
34	Glioma - Homo sapiens (human)[hsa05214]
	KRAS, CDK6,
35	Fatty acid elongation in mitochondria - Homo sapiens (human)[hsa00062]
	PPT2,
36	Primary immunodeficiency - Homo sapiens (human)[hsa05340]
	ZAP70,
37	Porphyrin and chlorophyll metabolism - Homo sapiens (human)[hsa00860]

	HMBS,
38	Bladder cancer - Homo sapiens (human)[hsa05219]
	KRAS,
39	Type II diabetes mellitus - Homo sapiens (human)[hsa04930]
	MAPK9,
40	SNARE interactions in vesicular transport - Homo sapiens (human)[hsa04130]
	STX11,
41	mTOR signaling pathway - Homo sapiens (human)[hsa04150]
	PDPK1,
42	Vibrio cholerae infection - Homo sapiens (human)[hsa05110]
	ATP6V1B2,
43	Hedgehog signaling pathway - Homo sapiens (human)[hsa04340]
	PTCH1,
44	Ether lipid metabolism - Homo sapiens (human)[hsa00565]
	CHPT1,
45	Basal cell carcinoma - Homo sapiens (human)[hsa05217]
	PTCH1,
46	Sphingolipid metabolism - Homo sapiens (human)[hsa00600]
	SPTLC1,
47	Drug metabolism - other enzymes - Homo sapiens (human)[hsa00983]
	TK2,
48	Wnt signaling pathway - Homo sapiens (human)[hsa04310]
	MAPK9, FRAT1,
49	Acute myeloid leukemia - Homo sapiens (human)[hsa05221]
	KRAS,

50	NOD-like receptor signaling pathway - Homo sapiens (human)[hsa04621]
	MAPK9,
51	Long-term potentiation - Homo sapiens (human)[hsa04720]
	KRAS,
52	RIG-I-like receptor signaling pathway - Homo sapiens (human)[hsa04622]
	MAPK9,
53	Adipocytokine signaling pathway - Homo sapiens (human)[hsa04920]
	MAPK9,
54	p53 signaling pathway - Homo sapiens (human)[hsa04115]
	CDK6,
55	VEGF signaling pathway - Homo sapiens (human)[hsa04370]
	KRAS,
56	B cell receptor signaling pathway - Homo sapiens (human)[hsa04662]
	KRAS,
57	PPAR signaling pathway - Homo sapiens (human)[hsa03320]
	PDPK1,
58	Small cell lung cancer - Homo sapiens (human)[hsa05222]
	CDK6,
59	Hypertrophic cardiomyopathy (HCM) - Homo sapiens (human)[hsa05410]
	TPM3,
60	Cardiac muscle contraction - Homo sapiens (human)[hsa04260]
	TPM3,
61	Renal cell carcinoma - Homo sapiens (human)[hsa05211]
	KRAS,
62	Regulation of actin cytoskeleton - Homo sapiens (human)[hsa04810]

	VCL, KRAS,
63	Arachidonic acid metabolism - Homo sapiens (human)[hsa00590]
	GPX1,
64	Glycerophospholipid metabolism - Homo sapiens (human)[hsa00564]
	CHPT1,
65	Adherens junction - Homo sapiens (human)[hsa04520]
	VCL,
66	Long-term depression - Homo sapiens (human)[hsa04730]
	KRAS,
67	Vascular smooth muscle contraction - Homo sapiens (human)[hsa04270]
	ADCY7,
68	Lysosome - Homo sapiens (human)[hsa04142]
	PPT2,
69	Spliceosome - Homo sapiens (human)[hsa03040]
	RBM25,
70	Jak-STAT signaling pathway - Homo sapiens (human)[hsa04630]
	STAM,
71	Axon guidance - Homo sapiens (human)[hsa04360]
	KRAS,
72	Tight junction - Homo sapiens (human)[hsa04530]
	KRAS,
73	Cell cycle - Homo sapiens (human)[hsa04110]
	CDK6,
74	Oocyte meiosis - Homo sapiens (human)[hsa04114]
	ADCY7,

75	Calcium signaling pathway - Homo sapiens (human)[hsa04020]
	ADCY7,
76	Leukocyte transendothelial migration - Homo sapiens (human)[hsa04670]
	VCL,
77	Purine metabolism - Homo sapiens (human)[hsa00230]
	AK4, ADCY7,
78	Huntington's disease - Homo sapiens (human)[hsa05016]
	GPX1,
79	Pyrimidine metabolism - Homo sapiens (human)[hsa00240]
	TK2,
80	Endocytosis - Homo sapiens (human)[hsa04144]
	STAM,
81	ECM-receptor interaction - Homo sapiens (human)[hsa04512]
	COL1A1, AGRN,
82	Oxidative phosphorylation - Homo sapiens (human)[hsa00190]
	ATP6V1B2,
83	Cytokine-cytokine receptor interaction - Homo sapiens (human)[hsa04060]
	CX3CR1,

Table S10. Targeted genes and KEGG pathways of hsa-miR-660

1	Peroxisome - Homo sapiens (human)[hsa04146]
	SCP2, FAR1, ACSL1,
2	Protein export - Homo sapiens (human)[hsa03060]
	SEC62, HSPA5,
3	Drug metabolism - other enzymes - Homo sapiens (human)[hsa00983]
	TK2, DPYD,
4	PPAR signaling pathway - Homo sapiens (human)[hsa03320]
	SCP2, ACSL1,
5	beta-Alanine metabolism - Homo sapiens (human)[hsa00410]
	DPYD,
6	Pantothenate and CoA biosynthesis - Homo sapiens (human)[hsa00770]
	DPYD,
7	Thyroid cancer - Homo sapiens (human)[hsa05216]
	TPM3,
8	Primary bile acid biosynthesis - Homo sapiens (human)[hsa00120]
	SCP2,
9	Notch signaling pathway - Homo sapiens (human)[hsa04330]
	LFNG,
10	Pyrimidine metabolism - Homo sapiens (human)[hsa00240]
	TK2, DPYD,
11	Prion diseases - Homo sapiens (human)[hsa05020]
	HSPA5,
12	Adipocytokine signaling pathway - Homo sapiens (human)[hsa04920]
	ACSL1,

13	Hypertrophic cardiomyopathy (HCM) - Homo sapiens (human)[hsa05410]
	TPM3,
14	Cardiac muscle contraction - Homo sapiens (human)[hsa04260]
	TPM3,
15	Dilated cardiomyopathy - Homo sapiens (human)[hsa05414]
	TPM3,
16	TGF-beta signaling pathway - Homo sapiens (human)[hsa04350]
	SP1,
17	Adherens junction - Homo sapiens (human)[hsa04520]
	VCL,
18	Fatty acid metabolism - Homo sapiens (human)[hsa00071]
	ACSL1,
19	Insulin signaling pathway - Homo sapiens (human)[hsa04910]
	RHOQ,
20	Parkinson's disease - Homo sapiens (human)[hsa05012]
	LRRK2,
21	Ubiquitin mediated proteolysis - Homo sapiens (human)[hsa04120]
	UBE2D1,
22	Spliceosome - Homo sapiens (human)[hsa03040]
	RBM25,
23	Antigen processing and presentation - Homo sapiens (human)[hsa04612]
	HSPA5,
24	Axon guidance - Homo sapiens (human)[hsa04360]
	SEMA4D,
25	Leukocyte transendothelial migration - Homo sapiens (human)[hsa04670]

	VCL,
26	Focal adhesion - Homo sapiens (human)[hsa04510]
	VCL,
27	Huntington's disease - Homo sapiens (human)[hsa05016]
	SP1,
28	Endocytosis - Homo sapiens (human)[hsa04144]
	ACAP2,
29	Regulation of actin cytoskeleton - Homo sapiens (human)[hsa04810]
	VCL,
30	Neuroactive ligand-receptor interaction - Homo sapiens (human)[hsa04080]
	RXFP1,
31	MAPK signaling pathway - Homo sapiens (human)[hsa04010]
	MEF2C,
32	Purine metabolism - Homo sapiens (human)[hsa00230]
	PDE7B,
33	Pathways in cancer - Homo sapiens (human)[hsa05200]
	TPM3,
